# Split‐Aperture Xolography – Linear Volumetric Photoactivation with Short Axial Dimension and Low out of Focus Excitation

**DOI:** 10.1002/advs.202416105

**Published:** 2025-05-31

**Authors:** Martin Regehly, Stefan Hecht

**Affiliations:** ^1^ Faculty of Engineering and Natural Sciences Technical University of Applied Sciences Wildau Hochschulring 1 15745 Wildau Germany; ^2^ Department of Chemistry Humboldt‐Universität zu Berlin Brook‐Taylor‐Str. 2 12489 Berlin Germany; ^3^ Center for the Science of Materials Berlin Humboldt‐Universität zu Berlin Zum Großen Windkanal 2 12489 Berlin Germany

**Keywords:** photoswitches, split‐aperture xolography, two‐photon absorption, vectorial diffraction, volumetric 3D printing

## Abstract

Spatially confined photo‐excitation with the lowest possible activation of the remaining volume is of central importance for high‐resolution high‐density optical data storage, fluorescence microscopy, 3D‐lithography, and 3D‐printing. Two‐photon absorption (2PA) enables such applications yet leads to slow processing speed due to the underlying non‐linear absorption process. Here, Split‐Aperture Xolography (SAX), is introduced which uses stepwise excitation of dual‐color responsive molecules to initiate a linear volumetric photo‐reaction process that is up to several orders of magnitude more efficient than 2PA. The capabilities of SAX are investigated in a scenario study for focusing systems with high numerical aperture (NA) using a Python implementation of vectorial diffraction theory. The intersecting half‐cones generated by the split illuminated entrance aperture of the objective reduce the axial focal spot size of the activation distribution by up to a factor of two compared to 2PA targeting the same electronic transition. A steep average decline of the activation probability with the fifth power away from focus is found for a wide range of directions. This is significantly better in comparison to 2PA and prevents that undesired out‐of‐focus excitation events sum‐up with subsequent irradiations. This approach is expected to be advantageous for volumetric methods at the nanoscale.

## Graphical abstract

Stepwise excitation of dual‐color responsive molecules in a novel intersecting half‐cone geometry provides excellent spatial resolution while retaining the high efficiency and thus speed of the overall process. The approach provides volumetric methods with an enhanced performance at the nanoscale.

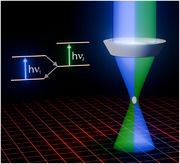

## 1 Introduction

**Figure 1:**
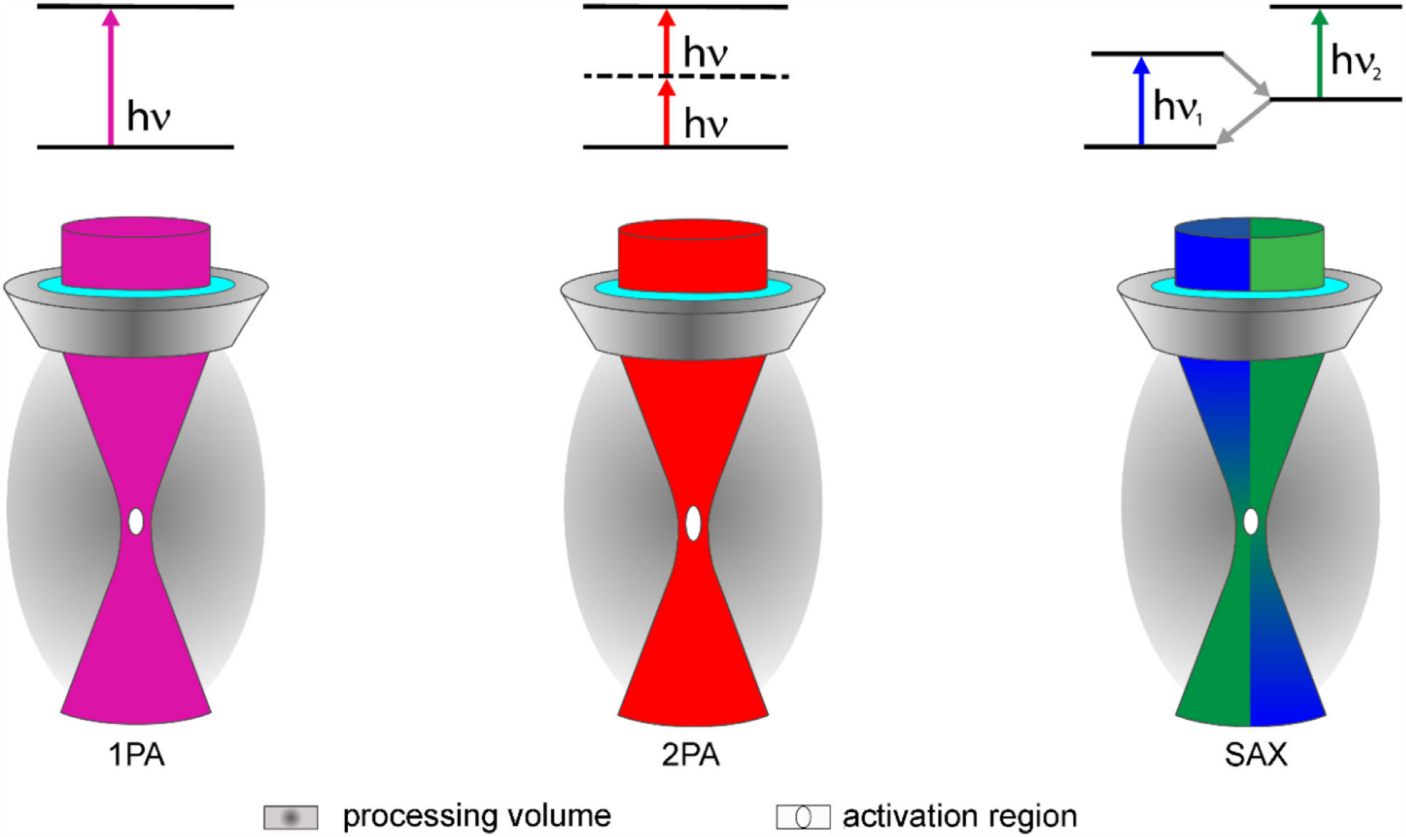
Photo‐activation of molecules in focused light beams due to one photon absorption (1PA), two photon absorption (2PA), and split‐aperture xolography (SAX).

Focusing light with controllable wavelength, spatial and temporal resolution has spawned a variety of applications, including photolithography, fluorescence microscopy, 3D‐printing, materials processing, and optical data storage.^[^ ^1^, ^2^, ^3^, ^4^, ^5^ ^]^ Extensive knowledge has been gained in recent decades to tailor intensity and polarization distributions within the focus, to facilitate optical trapping and super‐resolution imaging.^[^ ^6^, ^7^, ^8^, ^9^, ^10^, ^11^ ^]^ The primary way of activating molecules in a focal zone is based on single photon absorption (1PA) events, as shown in **Figure** **1**a. The linearity of this process implies that the distribution of the activated molecules corresponds to the illumination pattern within the spot under low absorption conditions.

Outside of the focal region, the irradiance decreases quadratically on average along the optical axis, as the area of the beam cone increases. This results in a significant background excitation, in case selective accumulation or localized expression of the absorbing species cannot be realized. For imaging of extended volumes, confocal techniques are required to eliminate out‐of‐focus contributions. In conjunction with super‐resolution techniques, they allow to realize lateral resolutions well below the diffraction limit.^[^ ^12^ ^]^ For data storage, nano‐fabrication, and structuring of materials in the 3D‐space, the problem remains and the 1PA‐process is not suited.

In case of two‐photon absorption (2PA), as depicted in Figure 1b, excitation occurs by simultaneous absorption of two photons, usually of half the energy of the optical transition, via an intermediate virtual state.^[^ ^13^ ^]^ The non‐linear process leads to a stronger confinement, the pattern of activated molecules is given by the squared illumination distribution within the focal region.^[^ ^14^ ^]^ This effect does not lead to a smaller voxel size in direct comparison to the 1PA method as indicated in Figure 1, since the wavelength used for the same transition is twice as long and the influence of the diffraction limit predominates. The non‐linear absorption probability in conjunction with the widening of the beam at a greater distance from the focus results in the excitation decreasing with the fourth power of the axial distance. This is one of the reasons for the success of 2PA with respect to two‐photon fluorescence microscopy, lithography, and material processing.^[^ ^15^, ^16^ ^]^ On the contrary, a significant limitation stems from the low cross‐sections of employed molecules for simultaneous two photon absorption on the order of σ_2*PA*_ = 10^−49^–10^−47^ cm^4^∙s∙photon^−1^ (10–1000 GM) whereas the linear absorption cross‐sections are in the range σ_1*PA*_ = 10^−18^–10^−16^ cm^2^∙molecule^−1^.^[^ ^17^, ^18^ ^]^ Since excitation is several orders of magnitude less probable, peak irradiances in the range of E = 10^12^ Wcm^−2^ are needed in the focal spot to activate enough molecules, that require low pico‐second or femto‐second lasers.^[^ ^15^, ^19^ ^]^ In case of two‐photon photopolymerization, process speeds are more than 1000 times lower at similar laser power compared to 1PA methods.^[^ ^20^ ^]^ If shorter wavelengths such as 515 nm are used for photopolymerization or pulse irradiances are above the TW/cm^2^ range, organic and inorganic precursor materials can be modified directly without the addition of 2PA absorbers.^[^ ^21^, ^22^, ^23^ ^]^ A route to increase the efficiency has been investigated based on energy transfer mechanisms such as triplet annihilation upconversion, in which a fluorescence with a shorter wavelength is generated after excitation, which in turn activates a photoinitiator.^[^ ^24^ ^]^

An alternative approach to confine the excitation to specific locations in the volume, but based on linear optical processes, is known as stepwise 2‐photon absorption.^[^ ^25^ ^]^ It has been employed to realize a fast volumetric 3D‐printing technique for macroscopic objects called xolography using photo‐switchable photoinitiator molecules, involving an intermediate ground‐state, and intersecting light beams of different wavelengths.^[^ ^26^, ^27^ ^]^ Later on, it was also applied to 3D nano‐printing based on photoinitiators using a stepwise singlet‐triplet activation mechanism possessing excited (triplet) intermediate states with typical lifetimes in the µs range.^[^ ^20^, ^28^ ^]^ This type of two‐color interaction with a photoresist, where both wavelengths must be present to trigger polymerization, enables fast and precise volumetric processing and is referred to as a synergistic system. Instead, two different photochemical reactions are triggered by each light color in orthogonal systems, which enables multi‐material printing. A third possibility is antagonistic systems in which the first wavelength activates the photoinitiator and the second deactivates it, which can be used to increase the resolution.^[^ ^29^ ^]^

In addition to abovementioned methods to confine the excitation volume, non‐linear chemical responses, such as the threshold effect during photopolymerization, can be exploited to achieve higher resolutions. By controlling the irradiance in particular, it is possible to control the voxel aspect ratio and generate structure widths that are below the diffraction limit in the range of 100 nm.^[^ ^1^, ^30^ ^]^

In this article, we explore a novel approach to superimpose two light beams of different wavelength predominantly for systems with a high numerical aperture (NA) as illustrated in Figure 1c, which dramatically enhances the achievable spatial resolution of xolography and extends its scope to different disciplines.

## 2 Results

### 2.1 Split‐Aperture Xolography (SAX)

We divide the entrance aperture of the objective into two halves, through each of which only the light of the first and second wavelength passes. As shown schematically in Figure 1c, the two light beams of different wavelengths cross primarily in the focal region. Dual color‐responsive molecules undergoing a sequential 1PA process are added to the entire processing volume.

According to the scheme $A\overset{\lambda_{1}}{⇌}B\overset{\lambda_{2}}{⇌}C$ the molecules in the half‐cone of the wavelength λ_1_ are excited from the ground state A to the latent state B. It has a finite lifetime due to a back reaction, which is a mandatory process to allow the return to state A in the absence of the second wavelength. Molecules in the intermediate state B absorb the light of the second half‐cone of wavelength λ_2_ that promotes them to the active state C. Due to the focusing arrangement, this transition is spatially confined to the intersection zone. Point‐by‐point writing generates the 3D‐activation pattern in which the desired response is mediated, such as light emission, solidification, or changes in specific material properties.^[^ ^31^ ^]^

### 2.2 Spatial Activation Distributions

In order to investigate the properties of the SAX concept and to derive generalized properties, we conducted a scenario study with parameters that are closely oriented to possible applications. In a first step the light distribution for each half‐cone in the focal zone is calculated, in this case also referred to as the point spread function (PSF). For this purpose, we developed a Python implementation for numerical computation of vectorial diffraction theory (see Experimental Section, containing also a link to publicly available script on Github), which is known to handle polarization effects and high numerical aperture systems precisely. In the second step, the activated pattern of molecules is given by the product of the individual PSFs due to the step‐wise absorption process.

Briefly, for focusing light in a homogeneous medium, the complex electric field amplitude vector **E** in the focal region can be calculated by the Debye‐Wolf integral, which for an aplanatic ideal lens satisfying the sine condition (approximated by a microscope objective to good extent) takes the following form:^[^ ^32^, ^33^, ^34^ ^]^

(1) $$\begin{array}{ccc} & & E\;\left(\begin{array}{c} \rho cos\varphi \\ \rho sin\varphi \\ z \end{array}\right)=\frac{-inkf}{2\pi }\;\int_{0|\pi }^{\pi |2\pi }\int_{0}^{\alpha }Q\\ & & \cdot \tilde{E}\left(\theta ,\phi \right)e^{ink\left[\rho \sin\theta \cos\left(\phi -\varphi \right)+z\cos\theta \right]}\sin\theta d\theta d\phi \end{array}$$

(2) $$Q=\frac{\sqrt{\cos\theta }}{2}\;\left(\begin{array}{ccc} \;\cos\theta +1+\left(\cos\theta -1\right)\cos2\phi & \left(\cos\theta -1\right)\sin2\phi & 2\sin\theta \cos\phi \\ \left(\cos\theta -1\right)\sin2\phi & \cos\theta +1-\left(\cos\theta -1\right)\cos2\phi & 2\sin\theta \sin\phi \\ -2\sin\theta \cos\phi & -2\sin\theta \sin\phi & 2\cos\theta \end{array}\right)$$

The illumination distribution is obtained from the formula PSF = c|**E**|^2^, where c is a constant, which we use to normalize the result as we are not interested in absolute values. The angular wavenumber is given by k = 2π/λ, the objective focal length is denoted by f. We have chosen λ_1_ = 405 nm as the first wavelength, which lies in the absorption band of many organic molecules such as fluorophores, photoinitiators, and photoswitches. 405 nm laser diodes are widely available, they had been developed for mass market applications such as blue ray players delivering stable optical power of up to several hundreds of mW. The second wavelength is set to λ_2_ = 515 nm and targets the absorption band of the latent state B of common two‐color responsive molecules as shown in Figure S1 (Supporting Information). Both visible wavelengths are generated from low‐cost laser diodes and can be guided with standard optical components.

**Figure 2:**
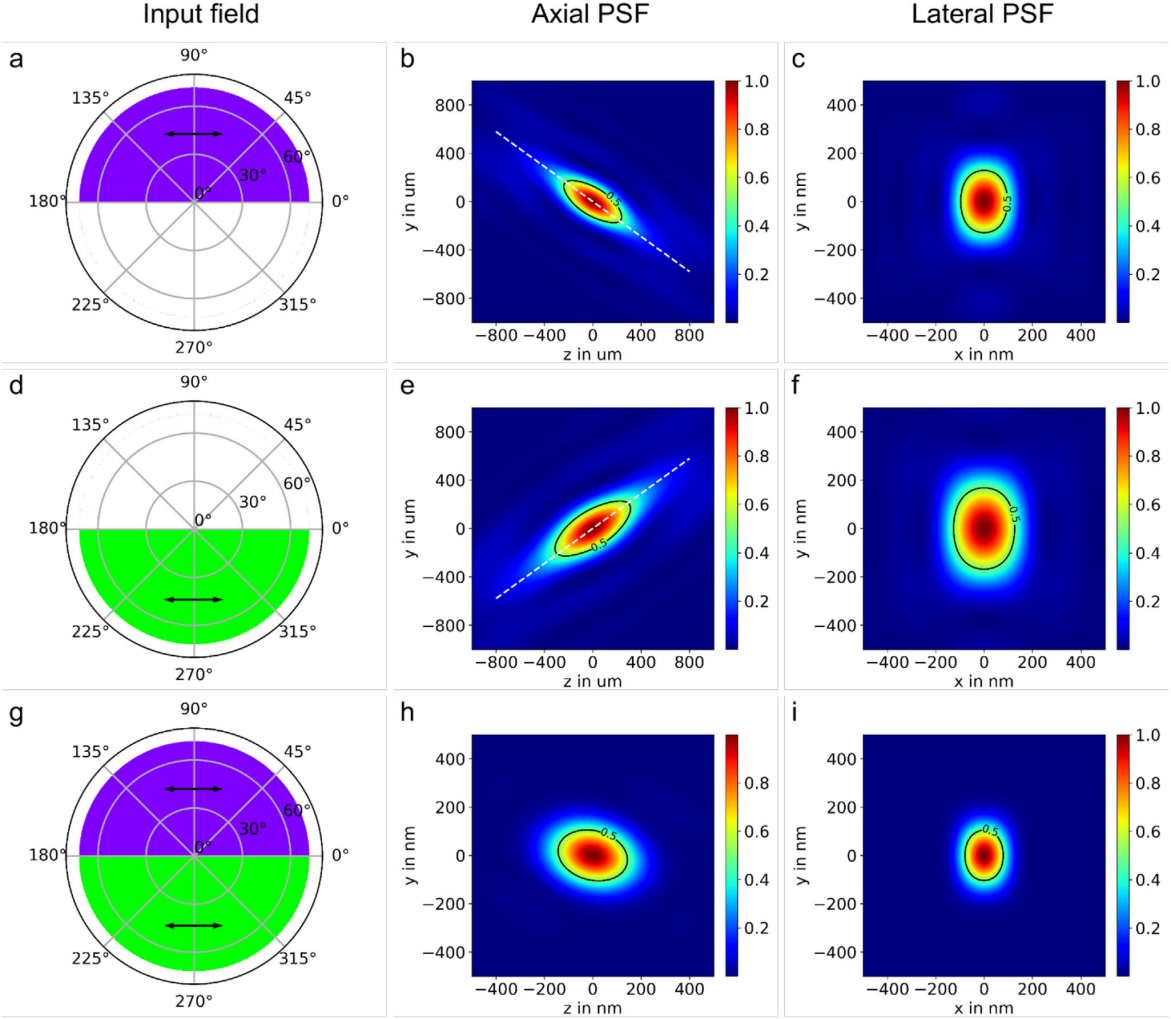
Split‐Aperture Xolography. First two rows: Axial and lateral illumination point spread function (PSF) for an upper half (405 nm) and lower half (515 nm) filled input aperture with x‐polarized (arrow) radiation. Third row: Axial and lateral activated molecule PSF for the combined input field.

Parameters of our calculation illustrated in **Figure** **2** include the refractive index n of the focusing media for the wavelength λ studied. For dual‐color techniques, the variation of n with wavelength must be taken into account, here we use the liquid monomer pentaerythritol tetraacrylate (PETA) as an example characterized by n(λ) = 1.44+16823.71 nm^2^/λ^2^.^[^ ^26^ ^]^ The semi‐angle of convergence of the lens is assumed to be α = 71.81°, representing an objective with high numerical aperture in the range of N_A_ = n sin α = 1.4–1.5 for the wavelengths studied. Numerical evaluation is performed by separately integrating over the upper and lower half of the circular input aperture, so the ϕ ranges from 0 to π and π to 2π, respectively. Each part of the entrance aperture of the objective is homogenously illuminated with linear polarized (x‐direction) collimated laser light as depicted in Figure 2a,d. In this case, the amplitude of the electric field across the whole input aperture is given by $\tilde{E}=\left[1,0,0\right]$. Figure 2b,e depicts the calculated axial illumination PSF in Cartesian coordinates for λ_1_ and λ_2_, respectively. The diagonal crossing of the focused half cones through the focal plane at z = 0 is clearly visible, as expected from the proposed concept. The intersecting angle of each cone with the z‐axis is given by α/2 and visualized by the dashed white line. In accordance with the diffraction limit, the expansion of the axial PSF at a wavelength of 515 nm is larger than at 405 nm. The same fundamental behavior is seen in the focal plane PSFs for λ_1_, λ_2_ in Figure 2b,c. As we are operating with half apertures for each wavelength, the effective N_A_ of the objective is reduced in the y‐direction, resulting in a greater extension of the lateral focus along this axis. However, the deliberately chosen x‐polarization of the input light counteracts the asymmetry of the lateral distribution, reducing its magnitude. We benefit here from a known mixing effect^[^ ^33^, ^35^ ^]^ of electric field components in high N_A_ systems which leads to an experimentally verified asymmetrical focus pattern^[^ ^36^ ^]^ for linear polarized input light fields and has already been used to fine‐tune structure sizes in 3D nanolithography.^[^ ^37^ ^]^ In Figure 2g both half apertures are illuminated, the super‐positioned focused light beams interact with molecules undergoing a two‐step absorption process, the resulting activation PSF is shown in Figure 2h,i. The axial and lateral distributions are significantly narrowed, below the diffraction limit with respect to the employed wavelengths. The slight asymmetry in Figure 2h is a consequence of the different sizes of the individual axial PSFs, it decreases with lower wavelength split.

### 2.3 Focal Spot Sizes

To check the validity of our calculation and compare our SAX results, we calculated the activation PSFs for the 1PA and 2PA processes using wavelength of 405 and 810 nm, i.e., half the photon energy, respectively. In this case numerical computation was performed with identical parameters but with fully illuminated entrance apertures (see Figure S2, Supporting Information). We determined the resolution by estimating the FWHM diameter from the respective spatial distribution and the values are summarized in **Table** **1**. The selected wavelength of 810 nm for the 2PA process enables a direct comparison with 1PA, but since common fs laser systems generate 515, 780, or 800 nm pulses, we have added the corresponding spot sizes in the table as well. The lateral resolution of SAX within the focal plane differs in the x,y directions because of the aforementioned asymmetry of the activation distributions. Further increase of the focal plane resolution is possible by directing a shift of the individual focal cones (see Figure S3, Supporting Information). Depending on the specific displacement, the lateral PSF can be narrowed below 100 nm in one direction (see Table 1 for FWHM diameters) while keeping the emerging side lobes below 25% compared to the central maximum.

**Table 1:** Axial and lateral spot diameters as calculated by vectorial diffraction theory for 1PA, 2PA, and SAX. Parameters: refractive index of the medium n = 1.54, 1.50, 1.47 (405, 515, and 780–810 nm), half‐angle of the objective α = 71.8°, input apertures are illuminated with collimated x‐polarized radiation. The estimated error is ≈3%, see the method section for details.

Method (Excitation wavelength)	Axial Spot Size FWHM in nm	Lateral Spot Size x,y FWHM in nm
1PA (405 nm)	347	191, 132
2PA (810 nm) (800 nm) (780 nm) (515 nm)	532 525 512 330	294, 200 290, 197 283, 192 182, 124
SAX (405 nm & 515 nm)	283	158, 207 98, 220^a)^

^a)^ displaced foci.

**Figure 3:**
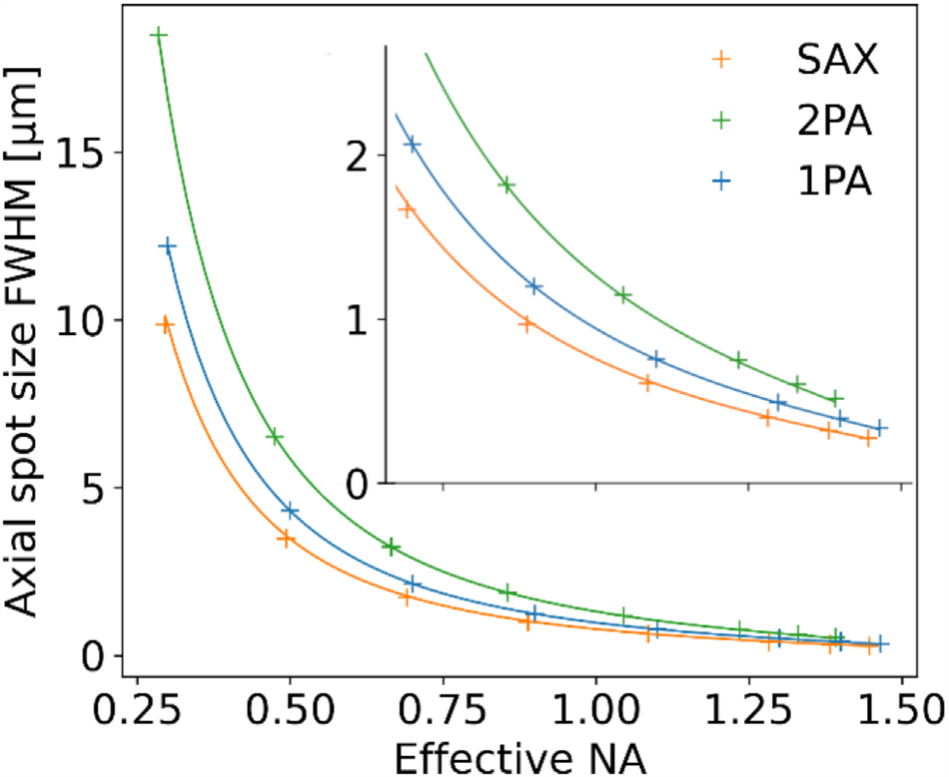
Axial focal spot size as a function of effective numerical aperture (NA) for SAX in comparison to 1PA and 2PA. Data‐points are obtained from simulations based on vectorial diffraction theory, solid lines are predictions from the closed form expressions of scalar diffraction theory.

Scalar diffraction theory proposes an axial diameter of 336 nm for the 1PA focus according to the formula (see methods below): ${\Delta z}_{\mathrm{FWHM}}^{1\mathrm{PA}}=\frac{0.443\lambda }{\mathrm{nsi}n^{2}\left(\alpha /2\right)}=\frac{0.886\lambda }{\left(n-\sqrt{n^{2}-NA^{2}}\right)}$ ^[^ ^38^ ^]^ The deviation of ≈3% from the determined value of 347 nm is attributed to the influence of polarization on the spatial distributions in systems with high NA.^[^ ^39^ ^]^ The axial diameter for 2 PA is given by ${\Delta z}_{\mathrm{FWHM}}^{2\mathrm{PA}}=\frac{0.319\lambda }{\mathrm{nsi}n^{2}\left(\alpha /2\right)}$ as derived in the method section, it is about a factor of $\sqrt{2}$ reduced in comparison to 1PA, however, due to the longer wavelength employed, it is of overall larger size. For SAX, the axial diameter of the focal spot of 283 nm FWHM is significantly narrowed in comparison to the reference 1PA and 2PA processes, supporting our proposed concept. To obtain a similar closed form expression for the axial resolution of the SAX process, we determined the FWHM spot sizes for a set of discrete NA values from the results of the Debye‐Wolf integral. The predictions for 1PA and 2PA (**Figure** **3**, solid lines) are based on the aforementioned equations by scalar diffraction theory and in very good agreement to our simulation, further indicating a low influence of polarization on the axial focus dimension for the system studied. For SAX, using two different wavelengths λ_1_ and λ_2_ with associated refractive indexes of the focusing medium n_1_ & n_2_, we found that ${\Delta z}_{\mathrm{FWHM}}^{\mathrm{SAX}}=\frac{0.319}{2\mathrm{si}n^{2}\left(\alpha /2\right)}\;\left(\frac{\lambda_{1}}{n_{1}}+\frac{\lambda_{2}}{n_{2}}\right)$ fits the simulated data points with precision and is essentially the result of averaging two 2PA processes utilizing different wavelengths through a common focusing objective with one‐half angular aperture α.

### 2.4 Out‐of‐Focus Activation

**Figure 4:**
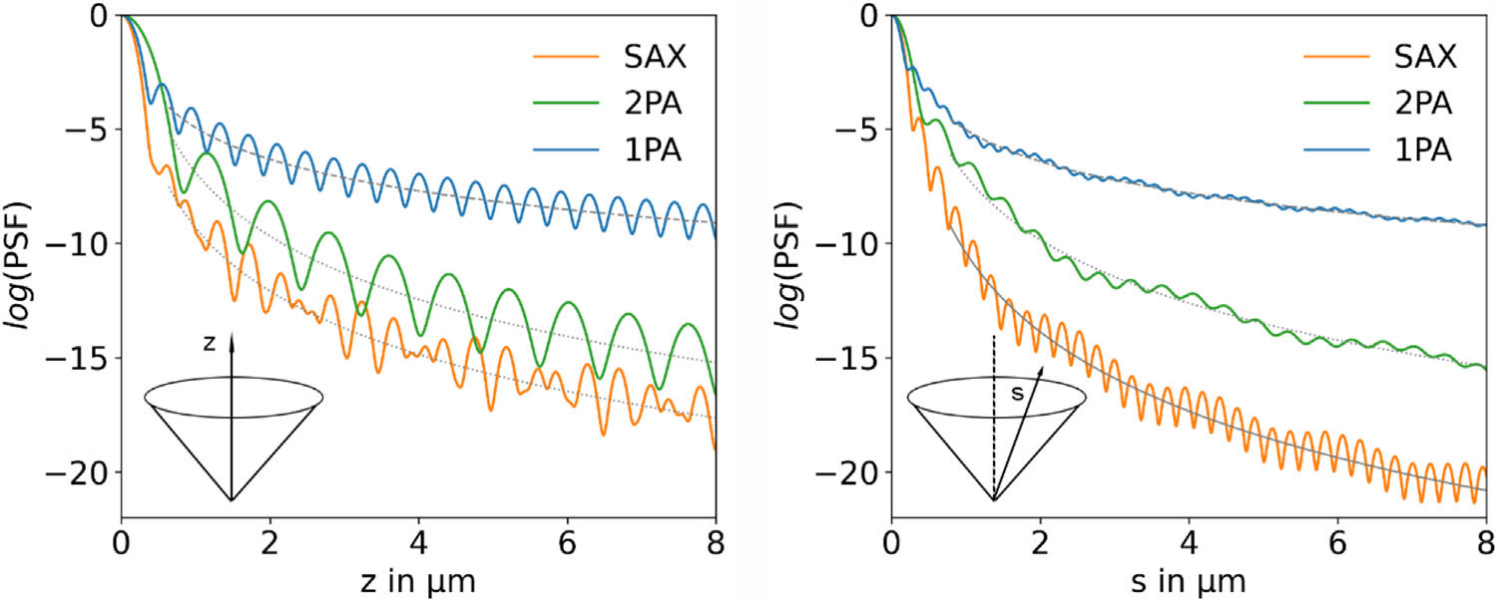
Decay of the point spread functions along optical axis z (left) and along a line *s* with slope of tan(α/2) in the z,y plane (right) depicting out‐of‐focus activation. Dashed, dotted, and solid fit curves represent the decrease in activation probability with distance from focus to the second, fourth, and fifth power.

We studied the PSFs along the optical axis starting from focus (z = 0) for the three high NA systems (**Figure** **4**a). The oscillations originate from the airy rings surrounding the central lobe, the spatial frequencies differ based on the wavelengths employed. In contrast to low NA focusing, the zero points of the axial profile disappear.^[^ ^40^, ^41^ ^]^ According to the known dependencies, the activation probability decreases quadratically and to the fourth power with distance for the 1PA and 2PA process, respectively. As indicated by the dotted fit curves, the 1/z^4^ behavior is also found for SAX which is reasonable as both half cones share the same optical path along the optical axis. The offset in the out‐of‐focus falloff between 2PA and SAX is caused by the latter's smaller axial focus diameter. The oscillatory waveform for SAX is determined by the product of the Airy ring patterns of the two wavelengths involved. Figure 4b shows the PSF values for a path *s* along α/2 emanating from focus, here we chose the z,y plane. This line corresponds to the propagating axis of the 515 nm half cone, depicted by the dashed line in Figure 2e. Findings are the same for 1PA and 2PA processes despite the lower magnitude in the airy ring oscillations. Instead for SAX, the activation probability decreases faster, with the fifth power of distance. This is due to the product of the PSFs: In the illuminated zone of the 515 nm half‐cone, the intensity drops on average quadratically along the path *s* under consideration further out from focus; at the same time, this is in the geometric shadow zone of the 405 nm half‐cone, where the decrease is greater with 1/s^3^. Tracing the activation distribution along a marginal ray at angle α in the z,y plane provides comparable results (see Figure S4, Supporting Information).

## 3 Discussion

In our disclosed approach, each half of the objective aperture generates an idealized half‐cone of light that overlaps in the focal zone. In combination with two‐color responsive molecules, SAX generates a subdiffraction activation pattern and leads to a significantly lower influence on the surrounding material volume compared to a fully illuminated aperture arrangement. The splitting induces an asymmetry of the activation distribution. In the axial direction it can be compensated by adapting the splitting ratio of the aperture, in the lateral dimension we minimize the influence by choosing a defined polarization direction under high numerical aperture conditions. Similar to the reference methods 1 and 2PA, shorter wavelengths and higher refractive indices of the material to be processed lead to a smaller activated voxel size.

One possibility of experimental realization is to generate two collimated half‐beams for each wavelength with the aid of apertures, which are combined via a dichroic mirror and directed into the entrance aperture of the focusing objective via a telescope as shown in Figure S5 (Supporting Information). Another alternative is to work with fully combined beams and insert a split spectral filter into the input aperture, with each half being transparent to the corresponding wavelength. The chromatically corrected standard objective is immersed (dip‐in) by the material to be processed. In case of stratified media, e.g. by using cover glasses, the theoretical calculation has to be adapted to the specific setup implemented. An initial alignment process is required for the spatial overlap of the two focal points, which can be controlled by observing the reflected light at optical interfaces, e.g. a slide with the material. Lateral adjustments are made by additional alignment mirrors in the beam path (not shown), while detuning the beam collimation can compensate for remaining axial chromatic shifts. Spatial refractive index fluctuations caused by inhomogeneities of the material or temperature variations are undesirable, as they could separate the overlapping focal points.

In general, our method offers a high level of flexibility to tailor the activation distribution, for example by dividing the aperture according to a different scheme, e.g. into an inner and an outer ring for each wavelength or by introducing spatially varying polarization patterns. As considered in this study, the half‐cones can be displaced with respect to each other reducing the activation spot size in one dimension.

SAX requires dual‐color responsive molecules. Their operating mechanism is based on two distinct sequential optical excitations, i.e., A→B and B→C, thus involving an intermediate species or state B. More precisely, the intermediate B can either be the ground state of a photogenerated species or a light‐induced electronically excited state. Photoswitchable molecules, such as spiropyranes as well as diarylethenes, among others, are particularly interesting because their photochromic properties can be adjusted over a wide range of the UV–vis spectrum and the lifetime of the photogenerated isomer ranges from tens of nanoseconds to minutes,^[^ ^42^ ^]^ allowing to generate sufficient local concentrations of the intermediate species B. Alternatively, molecules with excited singlet and triplet transient states, with typical lifetimes in the range of nanoseconds and microseconds, respectively, enable faster processing speeds yet require much higher light intensities to promote a sufficient amount of molecules into the transient state B.^[^ ^25^ ^]^ Well‐known representatives of this class that are used for radical photopolymerization are ketones, including benzil. For the studied systems with high numerical aperture, it has been demonstrated that continuous wave diode lasers of the mW‐class are sufficient to achieve the required focal intensities for the use of two‐color responsive molecules with microsecond transient lifetime.^[^ ^20^, ^28^ ^]^ The reverse reaction B→A has a profound influence on the lifetime of species B, it can be thermally driven or controlled with a third wavelength. The latter is an interesting option, as it enables a further increase in resolution and a reduction of the influence on the surrounding volume. A hurdle to practical implementation, especially for point‐by‐point processing schemes, is the presence of an undesirable, competing single‐wavelength activation channel in the dual‐color responsive molecules currently used.^[^ ^26^ ^]^ One reason lies in the remaining UV absorption band of the latent state B as depicted in Figure S1 (Supporting Information), which is observed in both, photoswitches and molecules based on a singlet‐triplet activation mechanism. In contrast to chromophores with a fixed structure, photoswitches offer promising potential of absorbing only the first wavelength in the initial state A and only the second wavelength in the intermediate state B, as their molecular structure changes during switching. With regard to future developments, the photoswitch must be designed in such a way that the short‐wave absorption band in state B has a significantly lower extinction coefficient or shifts spectrally to such an extent that UV excitation becomes unlikely. Dual color responsive molecules that have an intermediate electronic state such as the triplet state ideally do not exhibit photochemical reactions that originate from this state. Ketones such as benzil have a favorable low triplet state energy preventing bond cleavage within the surrounding molecular matrix but on the contrary abstract hydrogen atoms from their triplet ground state. This leads to the undesired formation of radicals when excited with the first wavelength in the UV range, and even the addition of scavengers, which quench the first excited triplet state, does not completely suppress this single wavelength initiation pathway.^[^ ^20^ ^]^

Depending on the structure of the molecule, photophysical or photochemical reactions related to the desired end application are mediated starting from the species/state C. For 3D‐printing two‐color responsive molecules have been reported based on photoswitchable^[^ ^43^ ^]^ and S_1_‐T_1_ functionality^[^ ^44^ ^]^ that induce a radical photopolymerization. Imaging techniques can be realized by utilizing photochromic molecules where the temporary state photogenerated by the first wavelength exhibits fluorescence emission under excitation with the second wavelength.^[^ ^45^, ^46^ ^]^ The addressing of small voxels with low z‐expansion and strong differentiation from surrounding areas with low energy input predestine the method for optical storage but biomedical applications and point‐of‐care diagnostics are also promising target areas.^[^ ^47^, ^48^ ^]^

A variety of other applications can be addressed through the use of photoswitchable molecules, which enable, among other things, modulation of the refractive index, changes in solubility, and mechanical and electrochemical properties.^[^ ^49^ ^]^

In conclusion, SAX is a conceptionally simple but powerful novel approach, preferentially for systems with high numerical aperture, with the advantage of a smaller axial spot size and lower background activation which can be realized with cost‐effective diode lasers and off‐the‐shelf optical components. We expect SAX to impact various areas of optical processing of materials from imaging over additive manufacturing to information processing.

## 4 Experimental Section

### Numerical Computation of Vectorial Diffraction Theory

Python scripts were developed that had been implemented in Jupyter Notebook and were made available on Github (see Supporting Information). The mathematical background and the details of the simulations performed is described in the following. The Debye‐Wolf integral according to Equation 1 was first transformed in order to be able to calculate the electric field in the focal zone numerically using the Python function libraries. In the calculation the entrance aperture is illuminated with a homogeneously, albeit arbitrarily, polarized collimated beam, i.e., this only considers plane waves with the complex Jones vector $\tilde{E}=\left(\tilde{E_{x}}e^{ifx},\tilde{E_{y}}e^{ify},0\right)$ where $\tilde{E_{x}}$ and $\tilde{E_{y}}$ denote the real valued field amplitudes and f_x_, f_y_ the phases. Using the abbreviation *g*  =  *nk*[ρsin θcos (ϕ − φ) + *z*cos θ] and the designation of the individual elements as a_11_, a_12_, …, a_33_ of the matrix Q (see Equation 2), the product $Q\cdot \tilde{E}$ in equation (1) was expanded. By further applying Euler's formula *e^iw^* = cos *w*  + *i*sin *w*, the double real integral of a complex‐valued function was divided into two separate double integrals:

(3) $$\begin{array}{ccc} & & E=\frac{-inkf}{2\pi }\;\int_{0|\pi }^{\pi |2\pi }\int_{0}^{\alpha }\frac{\sqrt{\cos\theta }}{2}\left(\begin{array}{c} \tilde{E_{x}}\;\cos\left(f_{x}+g\right)a_{11}+\tilde{E_{y}}\cos\left(f_{y}+g\right)a_{12}\\ \tilde{E_{x}}\;\cos\left(f_{x}+g\right)a_{21}+\tilde{E_{y}}\cos\left(f_{y}+g\right)a_{22}\\ \tilde{E_{x}}\;\cos\left(f_{x}+g\right)a_{31}+\tilde{E_{y}}\cos\left(f_{y}+g\right)a_{32} \end{array}\right)\\ & & \sin\theta d\theta d\phi +\frac{nkf}{2\pi }\int_{0|\pi }^{\pi |2\pi }\int_{0}^{\alpha }\frac{\sqrt{\cos\theta }}{2}\left(\begin{array}{c} \tilde{E_{x}}\sin\left(f_{x}+g\right)a_{11}+\tilde{E_{y}}\sin\left(f_{y}+g\right)a_{12}\\ \tilde{E_{x}}\sin\left(f_{x}+g\right)a_{21}+\tilde{E_{y}}\sin\left(f_{y}+g\right)a_{22}\\ \tilde{E_{x}}\sin\left(f_{x}+g\right)a_{31}+\tilde{E_{y}}\sin\left(f_{y}+g\right)a_{32} \end{array}\right)\\ & & \sin\theta d\theta d\phi \end{array}$$

To calculate the complex electric field amplitude vector in the focal region ***E *** =  (*E_x_*,*E_y_*,*E_z_*) at desired Cartesian coordinates (ρcos φ, ρsin φ, *z*), six double integrals were to be numerically evaluated. For this purpose, the dblquad^[^ ^50^ ^]^ function contained in the SciPy library for Python was utilized. Separated functions were defined in the script which were passed to dblquad that consecutively calculate g, the needed matrix components a_xx_, and the integrand value itself. The normalized intensity distribution in the focal zone which which was also refer as the point spread function was calculated from the electric field amplitude by *PSF*  =  *c*|*E_x_E_x_* * + *E_y_E_y_* * + *E_z_E_z_* *|, c was a normalization constant. Three different scripts were outcoupled for simplified computation of 1D axial PSF lineouts, 2D lateral (XY) focal plane, and 2D axial (YZ) PSF images. The parameters for the calculations used within this work were wavelength λ, refractive index n of the focusing media at λ, focusing angle α of the lens, the Jones vector of the electric field $\tilde{E}\;$ across the entrance aperture and an array of desired Cartesian coordinates for which the electric field in the focal zone should be computed. The script was comprehensibly programmed, depending on the number of coordinates to be calculated and the computer used, the runtime ranges from minutes to several hours. For the visualization of the PSF in Figure 2, a range of ±1 µm around the focus zone was calculated with a step size of 10 nm (200 × 200 electric field data points). PSF lineouts with a higher resolution of up to 0.5 nm were computed to determine the FWHM spot sizes. Taking into account the finite spatial grid spacing, the assumed wavelength uncertainty of ±1 nm, and a refractive index error of max. 5% lead to an error estimate of max. 3% for the calculated FWHM diameters.

### Axial Spot Sizes of 1 and 2PA Processes

An optical system consisting of a thin, circular lens focusing a plane monochromatic light wave of wavelength 𝜆 through a circular aperture into a medium of refractive index 𝑛 was considered. The lens has a numerical aperture *NA*  =  *n* *sin*α, where 𝛼 was the half‐cone focusing angle. The diffraction calculation in the scalar Debye framework yields the axial intensity distribution along z in the focal region according to $I\;\left(u\right)=I_{0}\;{|\frac{sin(u/4)}{u/4}|}^{2}$ using the normalized coordinate $u=\frac{8\pi nz}{\lambda }\;sin^{2}\left(\frac{\alpha }{2}\right)$.^[^ ^51^ ^]^ The distance from the focal point at which the intensity decreases to half (half‐max HM) could be determined by evaluating $I\;\left(u_{HM}/2\right)=\frac{I_{0}}{2}$, a numerical analysis provides a result of *u_HM_* =   ± 5.5663. By resolving the generalized coordinates, the full‐width half max (FWHM) axial spot diameter for the 1PA process is given by $\Delta z_{FWHM}^{1PA}=\frac{0.443\lambda }{nsin^{2}\left(\alpha /2\right)}$. Another representation is found by substituting $sin^{2}\;\left(\frac{\alpha }{2}\right)=\frac{1}{2}\;\left(1-\sqrt{1-sin^{2}\left(\alpha \right)}\right)$ and *sin*α  = *N_A_* /*n* which yields $\Delta z_{FWHM}^{1PA}=\frac{0.886\lambda }{\left(n-\sqrt{n^{2}-NA^{2}}\right)}$. In the low NA case, it can replace *sin* ^2^(α/2) ≈ α^2^/4 ≈  *NA* ^2^/4*n* ^2^ which gives $\Delta z_{FWHM}^{1PA}=1.77n\lambda /NA^{2}$.^[^ ^37^ ^]^ The axial intensity distribution for the simultaneous two‐photon excitation is obtained by squaring the distribution of the 1PA process to $I\;\left(u\right)=I_{0}\;{|\frac{sin(u/4)}{u/4}|}^{4}$. A similar analysis leads to the half‐maximum distances at *u_HM_* =   ± 4.0076 and thus for the FWHM spot size to the formula $\Delta z_{FWHM}^{2PA}=\frac{0.319\lambda }{nsin^{2}\left(\alpha /2\right)}=\frac{0.638\lambda }{\left(n-\sqrt{n^{2}-NA^{2}}\right)}$.

## Conflict of Interest

Both authors are co‐founders of xolo GmbH.

## Supporting information



Supporting Information

## Data Availability

The data that support the findings of this study are available in the supplementary material of this article. Python scripts are available on Github at the link https://github.com/RegehlyMartin/Vectorial_Diffraction_Calculation.git.
