# Supplementary material for: Split‐Aperture Xolography – Linear Volumetric Photoactivation with Short Axial Dimension and Low out of Focus Excitation
Source: Adv Sci (Weinh). 2025 May 31;12(31):e16105. doi: 10.1002/advs.202416105 (PMC12376575; doi:10.1002/advs.202416105)
Supplement: Supplementary file 1 — Supporting Information [file ADVS-12-e16105-s001.docx]

Supporting Information

Split-Aperture Xolography – Linear volumetric photoactivation with short axial dimension and low out of focus excitation

Martin Regehly* and Stefan Hecht*

^1^ Faculty of Engineering and Natural Sciences, Technical University of Applied Sciences Wildau, Hochschulring 1, 15745 Wildau, Germany.

^2^ Department of Chemistry, Humboldt-Universität zu Berlin, Brook-Taylor-Str. 2, 12489 Berlin, Germany.

^3^ Center for the Science of Materials Berlin, Humboldt-Universität zu Berlin, Zum Großen Windkanal 2, 12489 Berlin, Germany.

*Corresponding authors: [regehly@th-wildau.de](mailto:regehly@th-wildau.de) and [sh@hu-berlin.de](mailto:sh@hu-berlin.de)


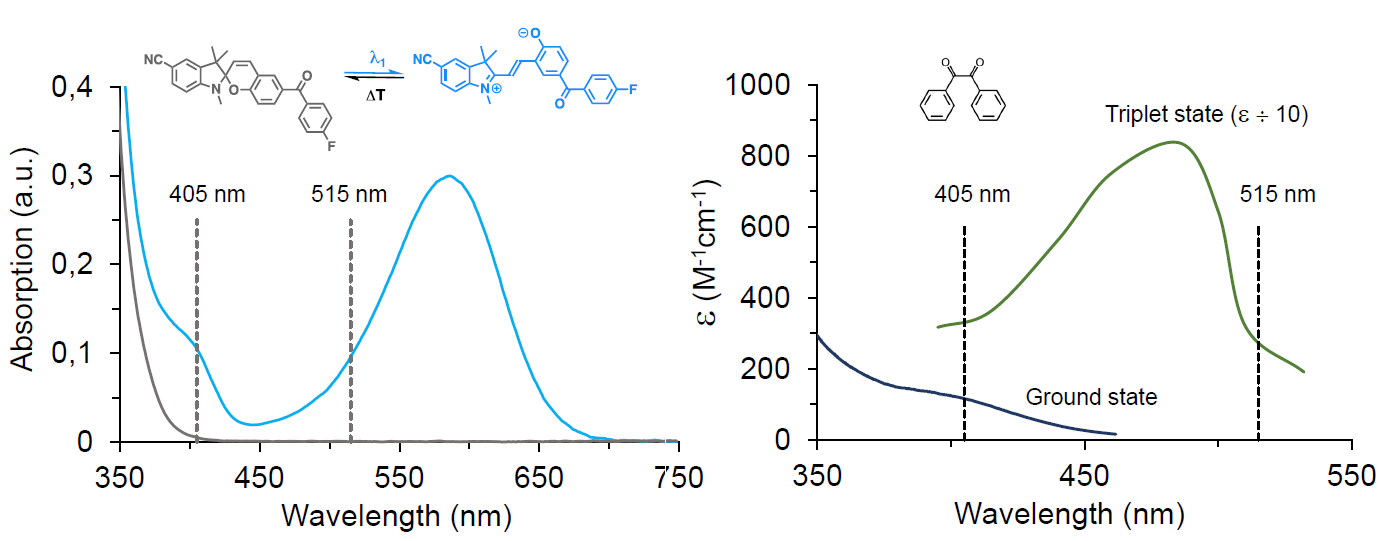


**Figure S1.** Absorption spectra of dual color photoinitiators (DCPI). Left: Photoswitchable DCPI dissolved in PETA, showing the spiropyran based ground state (gray) and latent state (blue) of the open merocyanine form. Data taken from [26] Right: Benzil allows a dual color activation mechanism involving a singlet ground state and a latent triplet state. Absorption spectra are reproduced from A. Bartecki et al., 1961 and T.-S. Fang et al., 1978.


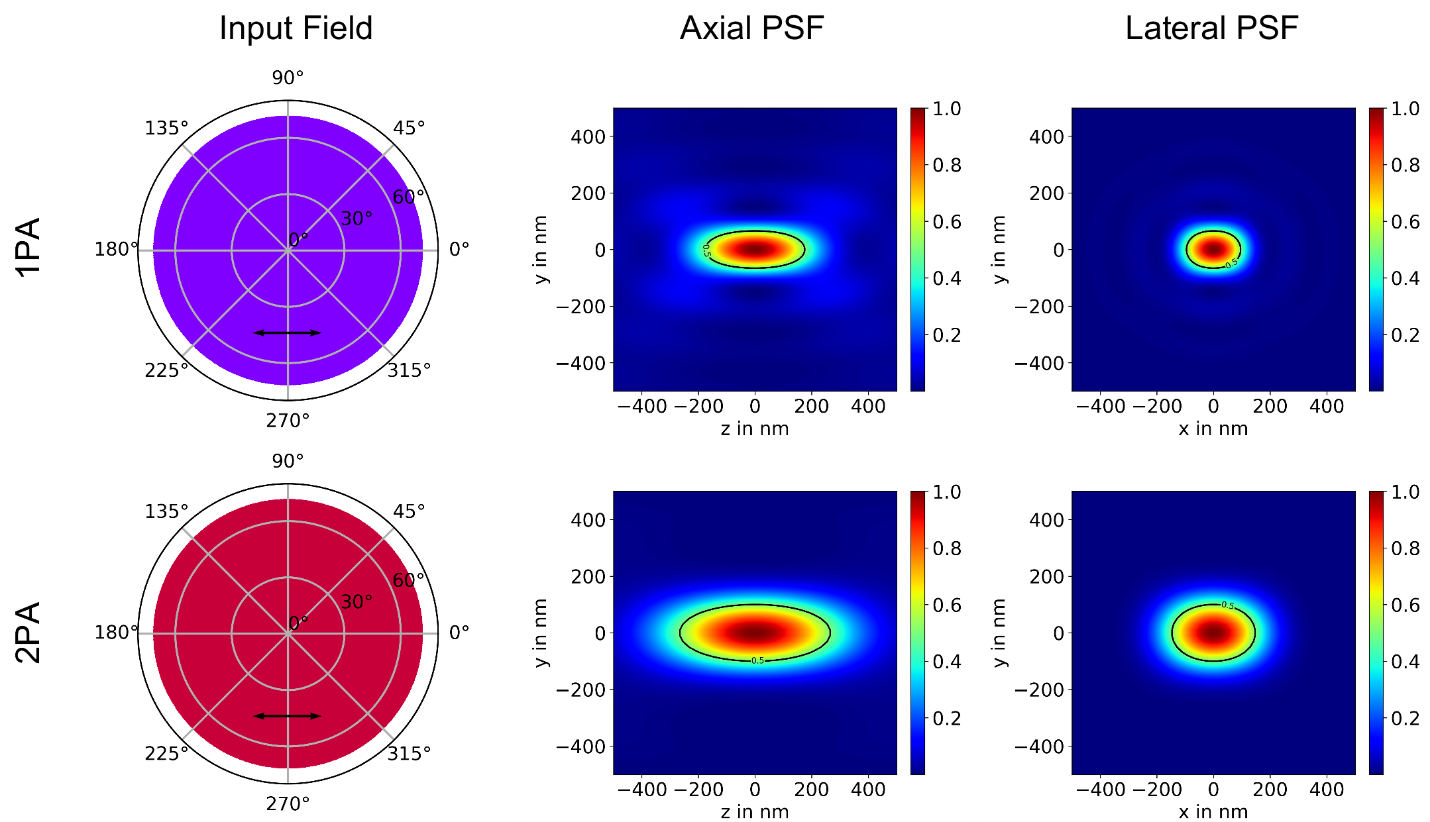


**Figure S2.** First row: Calculated activation PSFs for the 1PA process based on a fully, homogenously illuminated (405 nm) input aperture with collimated, x-polarized radiation. Second row: Activation PSFs for the 2PA process (810 nm) under the same overall conditions.


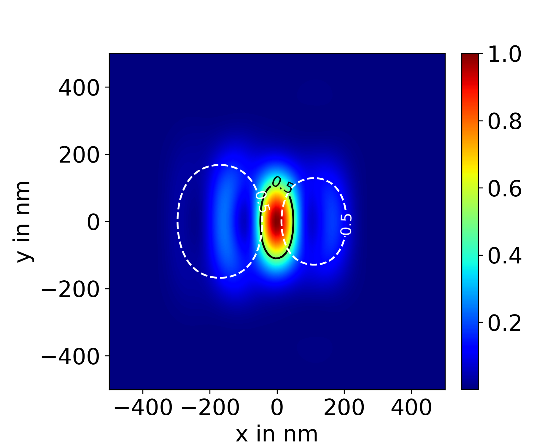


**Figure S3.** Lateral activation PSF for SAX in which both half-cone foci are shifted to achieve a minimum FWHM diameter (x) of 98 nm. Side lobes stay below 25% of the max. activation probability. Solid and dashed lines represent the FWHM of the resulting and overlaid initial distributions, respectively.


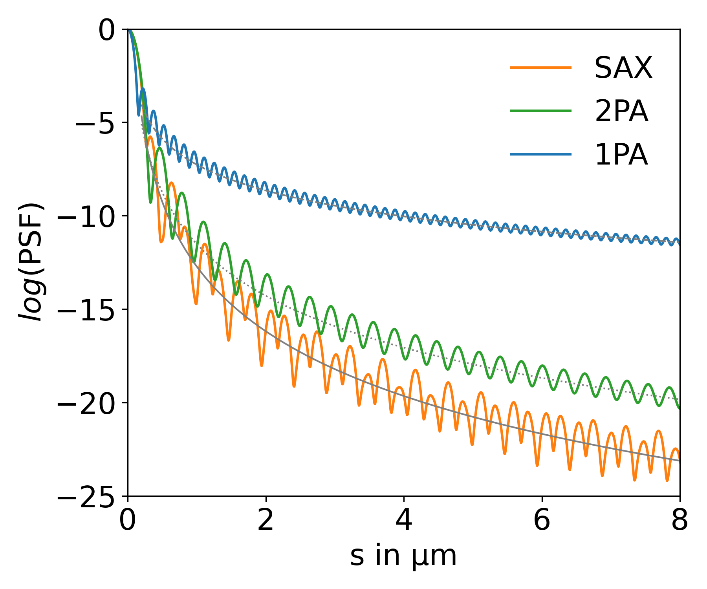


**Figure S4.** Line-out of the activation PSFs along a line *s* with slope of tan(α) in the YZ plane depicting out-of-focus activation. Dashed, dotted, and solid fit curves represent the decrease in activation probability with distance from focus to the second, fourth, and fifth power.


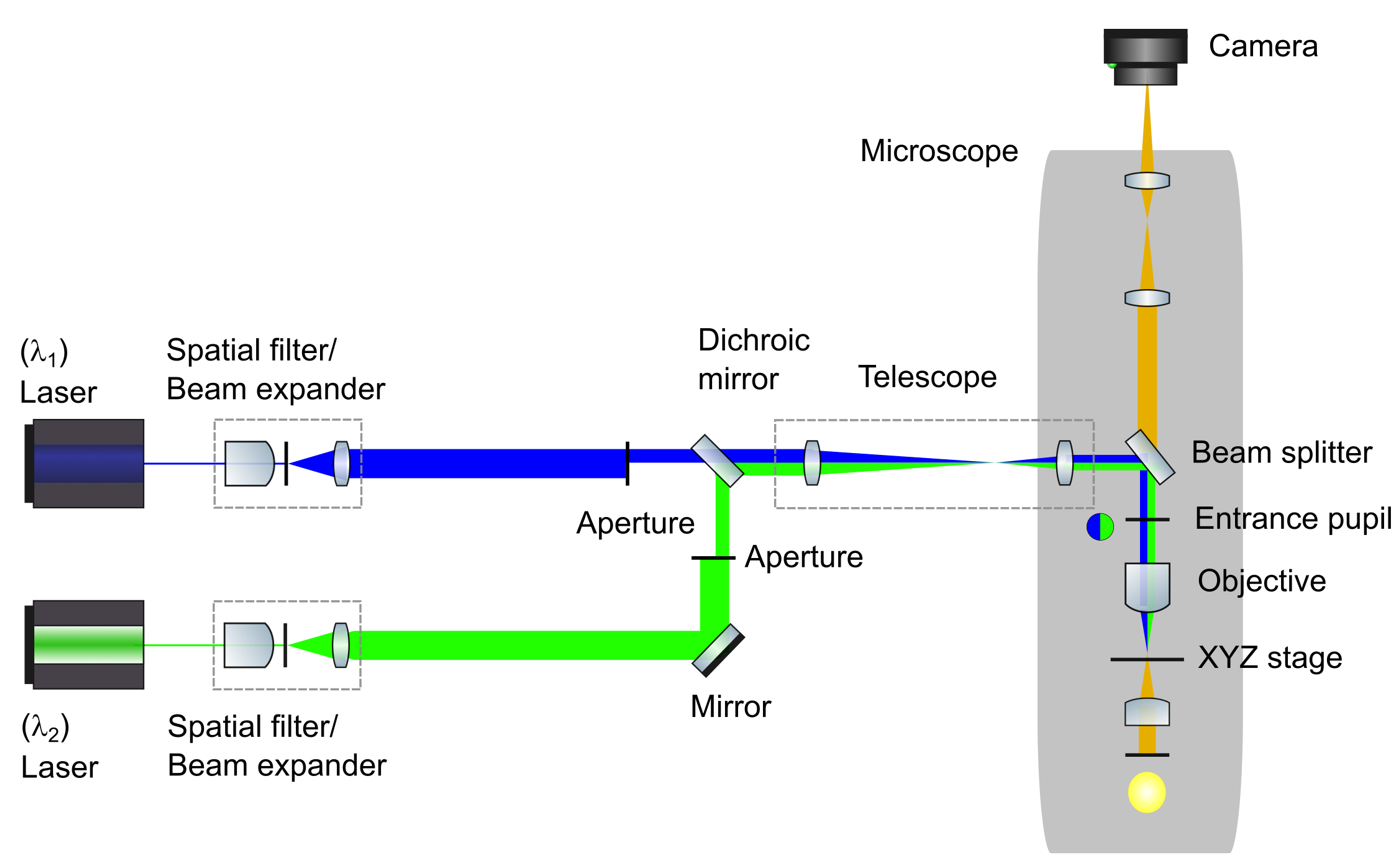


**Figure S5.** Proposed experimental setup for testing the SAX method. Two diode laser beams are expanded, followed by two inserted apertures and a dichroic mirror to create a collimated beam composed of both wavelengths. A telescope images the apertures in the entrance pupil of the apochromatic objective. The material to be processed is placed on an XYZ piezo stage in an immersion (dip-in) configuration.

Python scripts are available on Github at the link: <https://github.com/RegehlyMartin/Vectorial_Diffraction_Calculation.git>
